# Exploring Whether and How People Experiencing High Deprivation Access Diagnostic Services: A Qualitative Systematic Review

**DOI:** 10.1111/hex.14142

**Published:** 2024-07-15

**Authors:** Christine Vincent, Lee‐Ann Fenge, Sam Porter, Sharon Holland

**Affiliations:** ^1^ Department of Social Sciences and Social Work, Faculty of Health and Social Sciences Bournemouth University Bournemouth UK; ^2^ Department of Nursing Science, Faculty of Health and Social Sciences Bournemouth University Bournemouth UK

**Keywords:** barriers, deprivation, diagnostic services, diagnostics, facilitators, public involvement

## Abstract

**Introduction:**

To contribute to addressing diagnostic health inequalities in the United Kingdom, this review aimed to investigate determinants of diagnostic service use amongst people experiencing high deprivation in the United Kingdom.

**Methods:**

A systematic review was conducted using three databases (EBSCO, Web of Science and SCOPUS) to search studies pertaining to diagnostic service use amongst people experiencing high deprivation. Search terms related to diagnostics, barriers and facilitators to access and deprivation. Articles were included if they discussed facilitators and/or barriers to diagnostic service access, contained participants' direct perspectives and focussed on individuals experiencing high deprivation in the United Kingdom. Articles were excluded if the full text was unretrievable, only abstracts were available, the research did not focus on adults experiencing high deprivation in the United Kingdom, those not including participants' direct perspectives (e.g., quantitative studies) and papers unavailable in English.

**Results:**

Of 14,717 initial papers, 18 were included in the final review. Determinants were grouped into three themes (Beliefs and Behaviours, Emotional and Psychological Factors and Practical Factors), made up of 15 sub‐themes. These were mapped to a conceptual model, which illustrates that Beliefs and Behaviours interact with Emotional and Psychological Factors to influence Motivation to access diagnostic services. Motivation then influences and is influenced by Practical Factors, resulting in a Decision to Access or Not. This decision influences Beliefs and Behaviours and/or Emotional and Psychological Factors such that the cycle begins again.

**Conclusion:**

Decision‐making regarding diagnostic service use for people experiencing high deprivation in the United Kingdom is complex. The conceptual model illustrates this complexity, as well as the mediative, interactive and iterative nature of the process. The model should be applied in policy and practice to enable understanding of the factors influencing access to diagnostic services and to design interventions that address identified determinants.

**Patient or Public Contribution:**

Consulting lived experience experts was imperative in understanding whether and how the existing literature captures the lived experience of those experiencing high deprivation in South England. The model was presented to lived experience experts, who corroborated findings, highlighted significant factors for them and introduced issues that were not identified in the review.

## 1 Introduction

Diagnostic services are tests or procedures that identify diseases or conditions, allowing diagnoses to be made [1]. For non‐infectious diseases, increased time to diagnosis is associated with poorer outcomes [2, 3, 4, 5]. In high‐income countries, variations in stage of diagnosis and survival are associated with socioeconomic status, ethnicity, geographical location, age and other characteristics [6, 7, 8, 9, 10]. When differences are perceived as ‘unfair’ or ‘avoidable’, they are considered ‘health inequalities’ [11].

In England, diagnostic health inequalities relate to deprivation such that people experiencing high deprivation have higher levels of diagnosed illness on average than those in affluent areas [12, 13, 14]. However, the diagnostic health gap may be wider than some studies suggest. For instance, people experiencing high deprivation are less likely to participate in bowel, breast and cervical cancer screening than those in affluent areas, reducing the number of people experiencing high deprivation who receive diagnoses [15, 16]. One study found that despite higher illness rates for people in deprived groups, proportionately higher diagnosis was not observed [17].

This review aims to investigate determinants of diagnostic service use amongst British residents experiencing high deprivation. Portrayed views were synthesised to emphasise participants' lived experiences and explore factors that influence their decision to access diagnostic services.

## 2 Methods

This review uses ENTREQ guidelines for reporting on qualitative data synthesis [18] (Appendix A).

### 2.1 Patient and Public Involvement

Seven lived experience experts (LEEs) experiencing high deprivation in South England were recruited via purposive sampling by a gatekeeper (a local community‐based charity) and opportunity and snowball sampling by the researcher. Two patient and public involvement (PPI) sessions were held: one attended by a man in his 70s and a man in his mid‐20s, and one attended by two women aged 70+, a mother in her 20s and two women 50+. All participants live on the same estate and are White British. Once recruited, the LEEs contributed their lived experiences and personal expertise to enhance the applicability of the findings.

### 2.2 Defining Terms

For studies to be included in this review, they must have evaluated factors contributing to the active or passive decision‐making of individuals identified as having low socioeconomic status or experiencing high deprivation. In some cases, this was made explicit, while in others, the reviewers determined high deprivation levels based on data collected in the studies regarding income or educational level, occupational status and/or area of residence. This approach was driven by the way deprivation is measured in England, which incorporates factors such as income, education, employment and crime levels in a given geographical area [19].

### 2.3 Search Strategy

As detailed in protocol PROSPERO CRD42023399252, a comprehensive search was conducted using EBSCO, Web of Science and SCOPUS, searching MEDLINE, EMBASE, PsycINFO, CINAHL and SocINDEX. ClinicalTrials were searched for in The Cochrane Library and WHO Clinical Trials. Forward and backward citation searches were conducted for identified studies and generic web searches were conducted in Google Scholar using similar search terms. Grey literature was searched via the same methods and in the British Library's EThOS database. Searches were not restricted by language. The search period spanned from inception of the database to the present date. All searches took place from February 2023 to March 2023. The search strategy was repeated in November 2023 to identify literature published from March 2023. No papers were added after re‐running the search.

#### 2.3.1 Example Search Terms

Searches were limited to titles and abstracts and search terms related to diagnostics, barriers and facilitators to access and deprivation. A non‐exhaustive example of search terms used is included as follows:

- Diagnostic* OR screen*
- Barrier* OR obstacle* OR challeng* OR difficult* OR issue* OR problem*
- Facilitat* OR factor* OR influenc* OR enabl*
- “Low income” OR “low‐income” OR depriv* OR welfare OR poverty OR “socio‐economic” OR socioeconomic
- Utilis* OR utiliz* OR access* OR attend* OR “no show” OR “hard to reach” OR “no‐show” OR “hard‐to‐reach” OR (not N3 attend) OR (non N3 attend)

### 2.4 Inclusion and Exclusion Criteria

To ensure that included papers related to participants' perceptions of determinants of diagnostic service access in high‐deprivation areas in the United Kingdom, included papers must have discussed facilitators and/or barriers to diagnostic service access in the United Kingdom, contained participants' direct perspectives and focussed on British residents experiencing high deprivation. Due to this review aiming to emphasise the lived experience of participants, only qualitative studies and those incorporating PPI were included. Exclusion criteria included papers in which full text was unretrievable, abstracts only, those not focused on adults experiencing high deprivation in the United Kingdom, those not including participants' direct perspectives (e.g., quantitative studies) and papers unavailable in English.

### 2.5 Data Extraction

Identified records were initially stored on Zotero 6.0.30 [20]. Following duplication removal, C.V. screened titles and abstracts for eligibility. The remaining full‐text articles were screened according to eligibility criteria. To reduce bias, second reviewers (L.‐A.F., S.P. and S.H.) independently shared screening of 20% of the sample. Rates of concordance between first and second reviewers were high. Disagreements were resolved by discussion.

All included papers were uploaded in full to NVivo Pro 12.5 [21], where the data contained within ‘results’ and/or ‘findings’ sections were synthesised [22].

### 2.6 Quality Assessment

Critical Appraisal Skills Programme (CASP) was applied for this review [23]. Quality was assessed to inform analysis and synthesis. As no papers were assessed as low quality, none were excluded.

### 2.7 Data Synthesis

Inductive thematic synthesis was applied [19]. For a full description of the synthesis process, see PROSPERO CRD42023399252. In summary, three stages were followed: line‐by‐line coding of the findings, organising the codes into descriptive themes and inferring key analytical themes.

Initially, findings were entered into NVivo Pro 12.5 [21] and a code was associated with each line of text. Any data labelled as ‘finding’ or ‘result’ were included [22]. Next, codes were grouped together based on similarities and differences. If deemed necessary, new codes were added to capture group meaning. Finally, factors affecting study participants' decision to access diagnostic services were inferred from descriptive themes and codes. Analytical themes were organised into a conceptual model, as presented in Figure 2.

## 3 Results

### 3.1 Search Results

**Figure 1:**
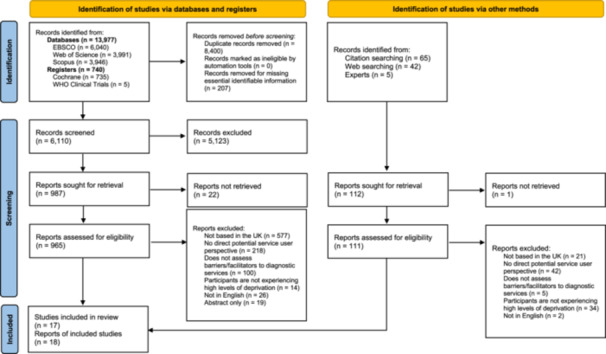
PRIMSA flowchart.

Initial searches identified 14,717 papers. After removing 8400 duplications and 207 for missing identifiable information (title or author names), 6110 were screened. During the first round of screening, 5123 papers were excluded for irrelevance to the review. As a result, 987 papers were sought for retrieval. A further 22 could not be retrieved. Therefore, 965 papers were assessed for eligibility. During full‐text screening, 954 studies were excluded for not meeting one or more inclusion criteria and 11 papers were included. Following backward and forward citation searching, an additional seven papers were identified. Therefore, 18 papers were included in the final review (Figure 1).

Table 1 describes the characteristics of each included paper.

**Table 1:** Summary of study characteristics.

Study	Participants	*N*	Methods used	Aim	Main outcome
Ali et al. [24]	Aged 50–75 years at high risk of developing lung cancer	748	Questionnaire with open‐ended question	To identify the barriers to participation among high‐risk individuals in the UK Lung Cancer Screening pilot trial.	Main reported reasons for screening non‐attendance were practical (e.g., travel issues, comorbidities, carer responsibilities, etc.) and emotional barriers (e.g., dislike for hospitals, low perceived personal risk and fear).
Brown et al. [25]	Aged 55+, smokers or ever smokers, living in deprived areas	16	Focus group and individual telephone interviews	To identify ways to increase opportunity for uptake of a lung cancer screening programme amongst people who could benefit most.	Several barriers and facilitators identified, relating to acceptability, awareness, reminders and endorsement, convenience and accessibility.
Dharni [26]	Aged 55–74 patients of GP practices in southeast London	50	Semi‐structured, one‐to‐one interviews	To explore the beliefs of Black African, Black Caribbean and White British people residing in a socio‐economically diverse area of southeast London about colorectal cancer screening participation.	Belief types in all ethnic groups that might serve to encourage screening participation: Belief that screening could save one's life, knowledge of someone with cancer, emotions and behavioural regulation.
Dharni et al. [27]	Aged 55–74 patients of GP practices in southeast London	50	Semi‐structured, one‐to‐one interviews	To explore the factors affecting colorectal cancer screening participation in an ethnically and socio‐economically diverse inner‐city population.	Lack of awareness of screening was a barrier for all. Cancer fear was a barrier for White British participants. Misunderstanding instructions was a barrier for people of low socio‐economic status (SES) regardless of ethnicity. Black African and Black Caribbean participants reported a perceived civic duty to participate in screening.
Goyder et al. [28]	Patients and practitioners of GP practices	72	Semi‐structured interviews	To examine perceived need for and provision of information before participation in a diabetes screening programme in English general practices.	Patients demonstrated a lack of understanding regarding benefits and disadvantages of diabetes screening and implications of screening results. Although letters were sent, these did not ensure that patients were better informed than those invited by telephone or opportunistically.
Hall et al. [29]	NHS Bowel scope screening programme attenders and non‐attenders from GP practices in northeast and east of England	73	Semi‐structured interviews and analysis of written accounts	To investigate responses to the screening invitation to inform understanding of decision‐making, particularly in relation to non‐participation in screening.	Key reasons for non‐attendance: Perceived or actual lack of need; inability to attend; anxiety and fear about bowel preparation, procedures or hospital; inability or reluctance regarding enema; low perceived susceptibility to bowel cancer; understanding of harms and benefits.
Hipwell et al. [30]	Regular and non‐regular screening attenders and primary care professionals and those who conduct screening	62	Multiperspectival, semi‐structured interviews	To examine the experiences of patients, health professionals and those who conduct screening; their interactions with and understandings of diabetic retinopathy screening; and how these influence uptake.	Facilitators for attendance: Knowledge about diabetic retinopathy and screening. Barriers to attendance: Psychological, pragmatic and social factors; confusion around how screening differs from a normal eye test; convenience and transport; pain and visual disturbance from the test.
Lindenmeyer et al. [31]	Patients, primary care professionals and those who conduct screening	62	Semi‐structured interviews	To identify factors contributing to high or low patient uptake of retinopathy screening.	Modifiable factors identified: Practice communication; contacting patients pre‐ and post‐screening; integration of screening with other diabetes care; focusing on the newly diagnosed; perceptions of non‐attenders. Non‐modifiable factors: deprivation level; diversity of ethnicities and languages; transport and access.
Logan and McIlfatrick [32]	Women living in socially deprived areas who accessed mobile cervical screening unit	48	Focus groups	To explore the experiences and perceptions of cervical screening among women from a socially deprived area in the United Kingdom.	Fear, embarrassment, stigmatisation, timing of appointments and childcare responsibilities reported as barriers. Practices are poorly tailored to meet the needs of the socially deprived.
Marlow et al. [33]	Women, aged 50–64 years, from lower socioeconomic and minority ethnic backgrounds	38	Focus groups	To explore barriers to cervical screening among women of screening age from hard‐to‐reach groups.	Women felt they had poor knowledge of screening; reasons for non‐attendance: discomfort and embarrassment, negative perceptions of health professionals, worry and trust in results, concern about procedure, idiosyncratic beliefs and extreme negative experiences. Some women did not receive letters or prompts to be screened.
Orton et al. [34]	People who had been invited to diabetic retinopathy screening but not made an appointment	32	Telephone interviews	To assess equity of access to diabetic retinopathy screening in a geographically and ethnically diverse population and determine predictors for poor uptake.	Barriers to attendance: Unfamiliarity with the condition, need for more information regarding condition and screening, forgetting to make an appointment, other commitments having higher priority and previous clear results.
Palmer et al. [35]	Socio‐economically diverse sample of people who had previously not participated in bowel cancer screening	128	Focus groups	To explore reasons for non‐uptake of bowel cancer screening and to examine reasons for subsequent uptake among participants who had initially not taken part in screening.	Barriers to attendance: Shame associated with completing the test, preference for completing test in practice rather than at home, feeling well. Subsequent participation facilitated by discussions with family and friends.
Pfeffer [36]	Ethnically diverse sample of women living in Hackney, a high‐deprivation area in London	146	Focus groups	To explore women's perceptions of breast cancer and the three approaches to encouraging early presentation.	Consensus in groups about what causes breast cancer was not reached. Candidacy perceived as linked to ethnicity. Women who believed early detection can cure cancer were more likely to participate in screening. Women who believed cancer is always fatal perceived screening as a waste of time. Screening was perceived as inconvenient due to competing priorities. Religious women preferred a female doctor but would put this aside for medical necessity.
Quaife et al. [37]	Smokers, ex‐smokers and never smokers, aged 40+	21	Closed survey and interviews	To compare smokers' beliefs about lung cancer screening with those of former and never smokers within a low SES sample, to explore the views of lower SES smokers and ex‐ smokers in depth and to provide insights into effective engagement strategies.	Interviewees supportive of screening in principle, but doubtful over its effectiveness in long‐term survival for ‘heavy smokers’. Smokers had a perception of blame and stigma that deterred them from participating in screening. Value of screening undermined by belief that lungs are untreatable and lung cancer is fatal.
Saidi, Sutton, and Bickler [38]	Women who accepted or declined routine breast screening invitation	913	Questionnaire with open‐ended questions	To report the reasons given for attendance or non‐attendance in women invited for breast screening for the first time.	Reasons for attendance: Wanting to know if cancer is present, peace of mind, opportunity, early detection, awareness of risk, sensibility and being invited. Reasons for nonattendance: Recently screened elsewhere, practical barriers, fear and low perceived risk.
Tonge et al. [39]	Smokers and ex‐smokers, aged 50–80 years	33	Semi‐structured focus groups	To explore with ever smokers the acceptability of targeted lung screening and uptake decision‐making intentions.	Screening seen as reassuring; perceived benefits influenced uptake; emotional barriers reduced likelihood of uptake, as did practicalities such as accessibility and perceptions of individual risk and stigma.
Tsipa [40]	Aged 60–74 years, previously received invitation to participate in bowel cancer screening	27	One‐to‐one, semi‐structured interviews	To investigate psychological and demographic variables' influence on the decisional process towards screening behaviour.	Perceived barriers: Stigma; awareness, attitudes and beliefs; inconvenience; feelings of disgust; fear; and gender and sociocultural influences. Perceived facilitators: Social influences; helping oneself; increased awareness; gratitude for the NHS; positive attitudes.
Woof et al. [41]	British‐Pakistani women from a low socio‐economic background	19	One‐to‐one, semi‐structured interviews	To explore the experiences that British‐Pakistani women encounter when accessing the NHS Breast Screening Programme.	Themes: Lack of confidentiality and independence in screening; appraisal of information sources, i.e., community information invaluable while NHS materials are inaccessible; and personal suppositions of breast screening, e.g., cultural misalignment of service and perceiving screening as symptomatic.

### 3.2 Data Synthesis

The aim of this analysis was to develop a conceptual model illustrating determinants of participants' decision to access diagnostic services. Three key themes were identified, composed of 15 subthemes (Table 2).

**Table 2:** Themes and subthemes identified.

Theme	Subtheme	Subtheme description
Beliefs and Behaviours	Perceived benefits	Perception, particularly amongst participants of diagnostic testing, that there are benefits to early detection of illness and/or that the benefits of testing outweigh the negatives.
	It's my responsibility	Sense of familial, civic or legal duty to participate in testing. Familial duty played a significant role, with some non‐participants claiming that if they had a family to test for, they would be more likely to access diagnostic services.
	Valuing the NHS	Particularly common amongst those from minority ethnic and migrant backgrounds. Sense of appreciation for the NHS, which tends to encourage participation in diagnostic testing. However, not wanting to waste NHS resources could also act as a barrier to access.
	Cultural factors	Also common amongst those from minority ethnic and migrant backgrounds but tending to act as barriers to access. Includes, for example, reluctance from British‐Pakistani women to reveal breasts during testing and that ‘seeking help, even when feeling unwell, is perceived as a weakness’ [40].
	Diagnostic Testing is unnecessary or unimportant	Perceiving diagnostic testing as unimportant or unnecessary due to, for example, believing an invitation letter is unimportant, culturally held beliefs influencing perception of the benefits of testing, overestimation of one's ability to detect illness within oneself and, particularly for home testing, removal from the clinical setting reducing perceived importance of the test.
	Low perceived personal risk	Feeling that one is at low risk for the condition in question, particularly due to lack of present symptoms, no family history of the condition, perception of a healthy lifestyle and misconceptions about causal factors.
Emotional and Psychological Factors	Stigma	Stigma was found to relate to the condition in question, the diagnostic process itself, lifestyle factors associated with the condition and fear of being ‘shunned’ [26, 27]. While fear of stigma extended to friends and family, it was also found that some non‐participants feared being ‘treated less sympathetically by medical professionals’; for instance, if lung cancer was found in smokers [39].
	Fear	Fears often related to receiving a diagnosis, waiting a long time to receive results and that treatment is unpleasant and futile. Fear was also found to be closely linked to fatalism.
	Vicarious experiences	Vicarious experiences are those that are had by other people but are recalled by the potential participant when deciding whether to access diagnostic services. These include knowing someone who became ill from the condition but survived, suffered greatly from the condition or from treatment, died from the condition or had a negative experience with the diagnostic test. Vicarious experiences could act either as a barrier or facilitator, depending on their influence on the potential participant.
	Convenience of eest	If the diagnostic test was perceived by the potential participant as convenient, this acted as a facilitator to access. However, if the test was perceived as inconvenient, then this factor acted as a barrier. Importantly, what is perceived by one person as ‘convenient’ is not always perceived so by another.
Practical Factors	Practical barriers	Includes testing difficulties associated with illness or disability, transport issues, unpleasantness of the test and discomfort or pain experienced during the test.
	Competing priorities	Includes carer and family responsibilities, work commitments and competing health priorities. Some people found that it was difficult to arrange appointments around busy schedules, while others claimed that they could not attend appointments due to carer or other familial responsibilities.
	Lack of knowledge or awareness	Lack of knowledge or awareness around the condition itself, benefits of early detection, test practicalities and causal factors tended to act as a barrier to access.
	Healthcare professionals' characteristics	Includes perception of healthcare professionals' character, mannerisms and level of empathy, as well as their insistence and encouragement for the potential service user to participate in diagnostic testing.
	Ability to trust	Includes both ability to trust in and build rapport with healthcare professionals, as well as trust in the wider NHS/healthcare ‘system’.

**Figure 2:**
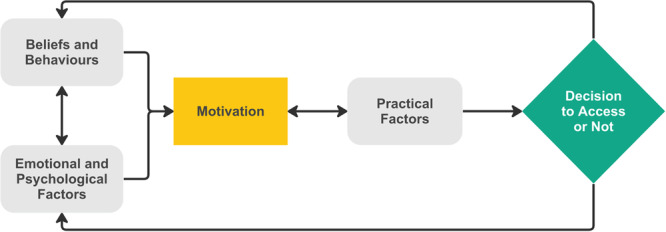
Conceptual model.

Themes were mapped into a conceptual model (Figure 2), which introduces three categories: Emotional and Psychological Factors, Beliefs and Behaviours and Practical Factors. Initially, Beliefs and Behaviours interact with Emotional and Psychological Factors to influence motivation to access diagnostic services. Motivation influences and is influenced by Practical Factors, contributing to a Decision to Access or Not. This decision influences Beliefs and Behaviours and/or Emotional and Psychological Factors and the cycle begins again.

The following sections expand upon the identified themes and sub‐themes. Quotes are derived from participants of included studies except where otherwise indicated.

### 3.3 Beliefs and Behaviours

**Figure 3:**
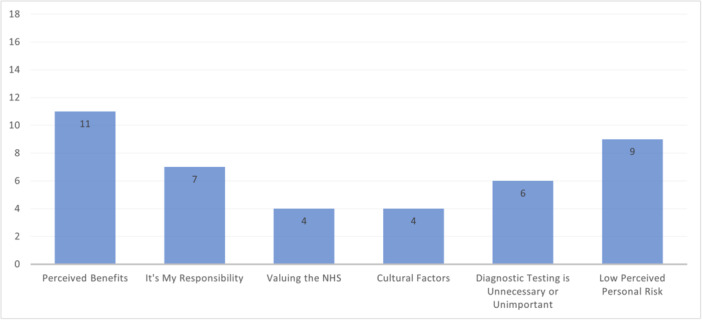
Number of papers discussing ‘Beliefs and Behaviours’ sub‐themes.

This theme is composed of perceived benefits, it's my responsibility, valuing the NHS, cultural factors, screening is unnecessary or unimportant and low perceived personal risk. The most discussed sub‐themes were ‘Perceived Benefits’ and ‘Low Perceived Personal Risk’ (Figure 3). The least discussed sub‐themes were ‘Cultural Factors’ and ‘Valuing the NHS’, likely because these sub‐themes relate to ethnic minority and migrant participants' views and few included studies highlighted explicit determinants for these groups.

#### 3.3.1 Perceived Benefits

For diabetes, diabetic retinopathy and lung, colorectal and breast cancers, the belief that early detection is beneficial [25, 26, 27, 28, 30, 35, 39, 40] and that benefits outweigh disadvantages [29, 33, 40] increased motivation to access diagnostic services.

Peace of mind was cited as a key benefit of early detection.

I like the fact that you instantly see and can get a decent steer on if there is anything negative; it's complete peace of mind—well my results anyway.

(Hipwell et al. [30])

Researchers in one study reported that, for some, the prospect of ‘more successful treatment and fewer complications’ that could ‘ultimately save one's life’ was a perceived benefit of early detection [27].

Even when testing was associated with negative connotations, participants believing in benefits felt that these outweighed disadvantages.

What's a few minutes of discomfort for our health, yeah.

(Marlow et al. [33])

#### 3.3.2 It's My Responsibility

In studies related to colorectal cancer and lung cancer, a sense of responsibility to access diagnostic services was a common facilitator [26, 27, 29, 39, 40].

Some participants claimed that familial responsibility encouraged them to access diagnostic services. In some cases, participants desired remaining healthy so they could continue to support their family [26, 39, 40], while others desired not to burden them with an ill family member [26, 27, 40].

I suppose really it's for your own peace of mind, isn't it? Plus the family, you know. I'm still married and have a couple of kids and grandchildren, you know, so it's not only that will sort of go, it's going to affect the family as well.

(Dharni [26])

#### 3.3.3 Valuing the NHS

Common amongst minority ethnic and migrant participants was appreciation for the NHS, primarily because it is free at point of use, does not discriminate against rich or poor and provides high quality of care [26, 27, 40].

NHS services were often contrasted to the healthcare system of the home country [26, 27, 36].

Those of us who have the privilege of being in this country, are lucky with the care and technology. Where I come from, Nigeria, you don't have these. People dying of one thing or the other … the state doesn't have any provision for them, so they die.

(Dharni and colleagues [26, 27])

However, in Pfeffer's [36] study, native British women were, according to researchers, ‘more likely to understand healthcare, including the [NHS Breast Screening Programme], as a citizenship right and to complain about having to pay for things like medicines and glasses’.

Participants from ethnic minority or migrant backgrounds also emphasised not wanting to waste NHS resources [26, 27, 29]. However, this was also cited as a barrier for some [25, 29].

I won't have treatment for cancer … So, you know, I just think I'm not wasting people's, the NHS's, money or whatever, you know, I'm just not.

(Hall et al. [29])

#### 3.3.4 Cultural Factors

For ethnic minority and migrant participants, cultural barriers were cited as deterrents to access [26, 27, 40, 41].

For instance, in Woof et al.'s [41] study on breast cancer screening, British‐Pakistani women were reluctant to reveal their breasts during testing, due to cultural values.

… she's just saying, her religion, says the same, her culture, if you're a daughter, you can't be uncovered.

(Woof et al. [41])

In Tsipa's [40] study on colorectal cancer, participants believed, according to the researcher, that ‘seeking help, even when feeling unwell, is perceived as a weakness’.

#### 3.3.5 Diagnostic Testing Is Unnecessary or Unimportant

In some studies, participants perceived diagnostic testing as unimportant or unnecessary, which reduced motivation to access [28, 29, 33, 41]. One participant in Goyder et al.'s [28] study on diabetes screening stated,

“[I tend not to] bother with letters … I'll see how important it is, if it's important I'll deal with it, if it's not that important then I'll leave it.”

Woof et al. [41] found that British‐Pakistani women viewed breast cancer screening as a symptomatic service, rather than an early detection or preventative measure.

… it's really when they get the disease and they feel it's [the breast] hard, then they will go, before that they think they don't need to go.

(Woof et al. [41])

Some perceived diagnostic testing as unnecessary, due to an overestimation of ability to detect cancer within the self [30]. However, in some cases, participants already received screening elsewhere or had been told by their doctor that screening was unnecessary for them [24, 29, 33, 38].

#### 3.3.6 Low Perceived Personal Risk

Low perceived personal risk, which tended to reduce motivation, was associated with breast, cervical, colorectal and lung cancers [24, 29, 33, 35, 38, 39, 41].

Common reasons for this belief were a perceived healthy lifestyle [24, 29, 35], being asymptomatic or normalising symptoms [26, 35, 38, 39, 40] and having no family history of the condition [33, 35].

I do not feel at risk of bowel disease because I am not a heavy drinker, I hardly ever take pills and I have been vegetarian for 25 years and have an excellent diet and fitness regime. But I still think it's a great idea to offer this screening to people 55+.

(Hall et al. [29])

### 3.4 Emotional and Psychological Factors

Emotional and Psychological Factors could positively or negatively impact motivation to access diagnostic services and include stigma, fear and vicarious experiences.

**Figure 4:**
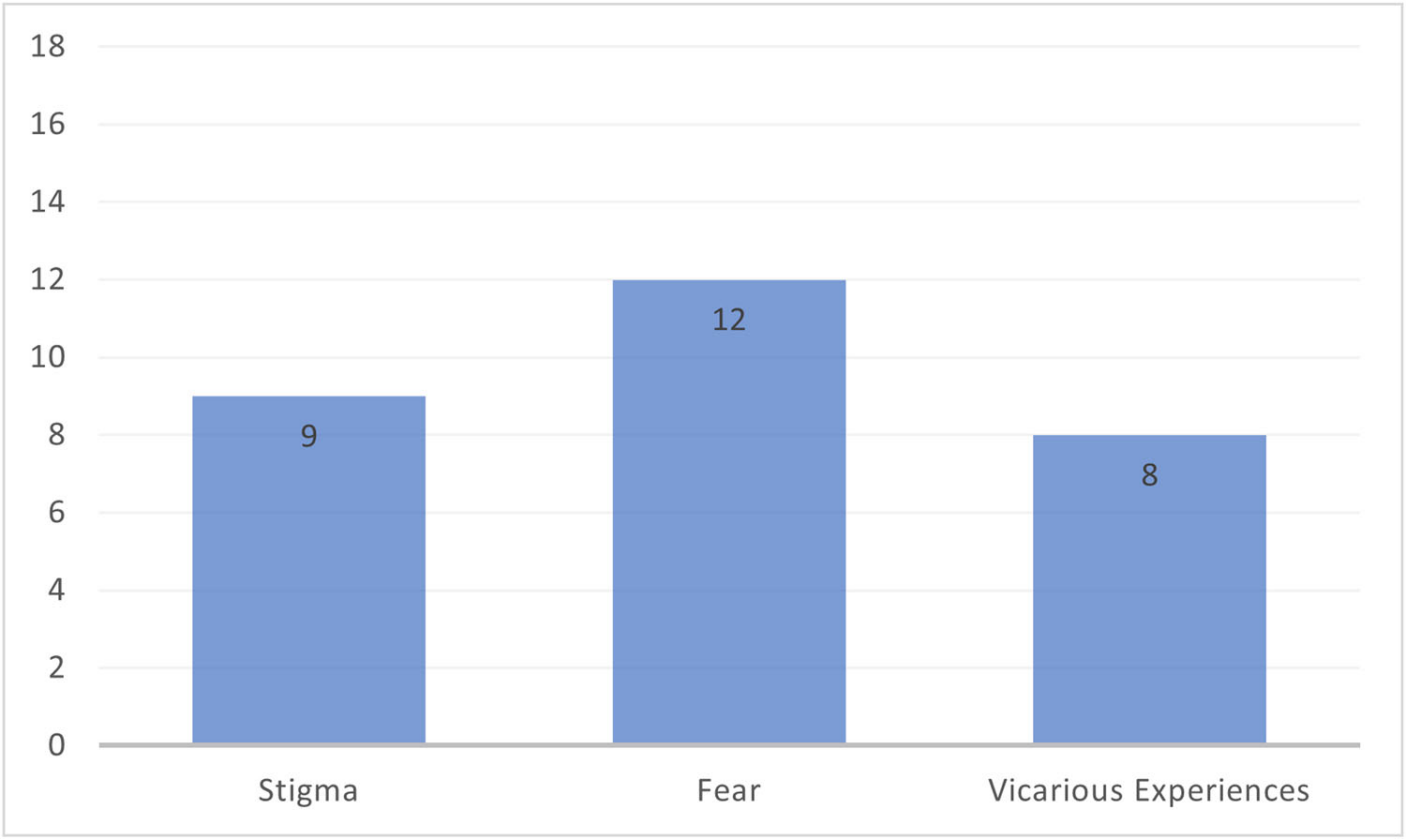
Number of papers discussing each ‘Emotional and Psychological Factors’ sub‐theme.

The most discussed subtheme within this theme was ‘Fear’ (Figure 4). ‘Stigma’ was discussed in half of the included studies, while ‘Vicarious Experiences’ was discussed in just under half.

#### 3.4.1 Stigma

Stigma, which tended to reduce motivation to access diagnostic services, was associated with cervical, colorectal and lung cancer [25, 26, 27, 32, 35, 39, 40]. Participants expressed concern over stigma related to the condition [26, 27, 40], lifestyle factors associated with that condition [25, 32, 39] and fear of being ‘shunned’ [27].

Some participants expressed concern that if lung cancer was found, they would be ‘treated less sympathetically by medical professionals’ [39].

For other conditions, such as colorectal cancer, anticipated stigma was associated with the ‘taboo’ nature of the test.

The problem is you wouldn't bring it up in the middle of a dinner party, you know … I find it awkward and embarrassing. It's not […] ‘I went down to the hospital and had a blood test today.’ That probably have more chance of entering a conversation than ‘I did this poo test’.

(Tsipa [40])

Closely linked to stigma was feelings of embarrassment, particularly for cervical and colorectal cancers [29, 32, 33, 35, 40].

#### 3.4.2 Fear

Fear could either increase or decrease motivation, based on what is feared. Most fears related to receiving a diagnosis of the condition [25, 26, 27, 35, 36, 37, 38, 39, 40], the screening procedure itself [29, 32, 35, 37, 39], waiting a long time to receive results [32, 39, 40] and unpleasantness and futility of treatment [35, 39, 40].

Fears were also expressed through fatalism, where participants believed that the condition was associated with ill health and death [33, 36, 37, 39, 40]. Those with fatalistic views tended not to access diagnostic services, viewing it as a ‘waste of time’ or not wanting to know if they had the condition [33, 36, 40].

Palmer et al. [35] found it common for participants to describe treatment as unpleasant and futile. For lung and colorectal cancers, there were fears that treatment would be ‘detrimental’ to quality of life [37].

Well would you like a bag stuck to you? And it's permanent as well. Just horrendous, I wouldn't be able to cope with that.

(Tsipa [40])

#### 3.4.3 Vicarious Experiences

Vicarious experiences are those experienced by others and recalled by the participant. These can be individuals who suffered or died from the condition [26, 27, 29, 30, 35, 39, 40] or had negative experiences with the procedure [32, 35].

Vicarious experiences sometimes related to knowing someone who suffered from the condition, which contributed to fear of receiving a diagnosis and, in some cases, that treatment is futile.

Sometimes all these treatments and nothing works, so I think I would just give in at the first hurdle … they (friends with bowel cancer) went through all that battle and nothing worked.

(Hall et al. [29])

In other cases, participants feared suffering, which improved motivation to access diagnostic services.

I had a very close friend who died of it, we were for many years close. So, and erm, I saw the whole process as such, I was with him throughout the period until he passed away … When you've seen someone close going through that process, then you understand why you fill in those, do those tests.

(Dharni et al. [27])

### 3.5 Practical Factors

Practical Factors could act as barriers or facilitators to participation and include convenience of testing, practical barriers, competing priorities, lack of knowledge or awareness, healthcare professionals' characteristics and ability to trust.

**Figure 5:**
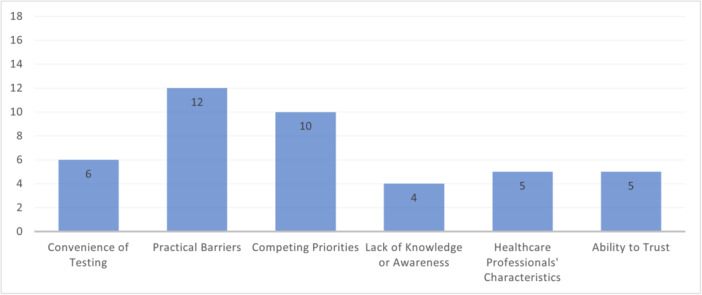
Number of papers discussing ‘Practical Factors’ sub‐themes.

The most discussed sub‐themes were ‘Practical Barriers’ and ‘Competing Priorities’, likely due to large variations in the type of practical barrier and competing priority (Figure 5).

#### 3.5.1 Convenience of Testing

For cervical, lung and colorectal cancers and diabetic retinopathy, perceived convenience of the procedure acted as a facilitator to access [25, 26], while perceived inconvenience acted as a barrier [30, 32, 33, 40].

Inconvenience ranged from appointment times and testing location [32, 33] to difficulties coping with aspects of the test, such as mydriasis drops for diabetic retinopathy screening [30]. Contrasting the convenience of home testing cited by Dharni [26], researchers in Tsipa's [40] study on colorectal cancer reported that home testing was perceived as inconvenient, due to ‘the screening process, including the manual handling and sampling of one's own stool, storing the kit in one's home and posting the kit to the laboratory for examination’.

#### 3.5.2 Practical Barriers

Practical barriers, which tended to reduce likelihood of access, even in the presence of motivation, included difficulties associated with illness or disability; transport issues; unpleasantness of the test; lack of appointment availability; and discomfort, embarrassment, or pain during testing [24, 25, 26, 27, 29, 30, 31, 32, 33, 35, 38, 40].

A common practical barrier was transport issues, particularly for diabetic retinopathy testing, which involves mydriasis drops being administered.

I felt I was blinded temporarily and got into a taxi and then got out of the car somehow. I had to cross the road and I was just looking like that [stares blankly] because I was waiting for the taxi and I had to do like that [waves arms].

(Hipwell et al. [30])

Additionally, issues such as travel distance, lack of public transport, rurality, journey cost, hospital parking or not having a car were prominent concerns [24, 25, 29].

Unpleasantness of the test deterred those invited to attend cervical or colorectal diagnostic appointments and those asked to complete at‐home tests [26, 29].

It's just such a horrible thing to have done.

(Marlow et al. [33])

Beyond unpleasantness, some participants expressed that the test may be painful, particularly for cervical screening [33].

#### 3.5.3 Competing Priorities

Competing priorities were factors that tended to reduce likelihood of access, even in the presence of motivation. Common competing priorities were carer or family responsibilities, work commitments and competing health priorities [24, 26, 27, 29, 30, 32, 33, 35, 38].

Inability to attend appointments due to family or carer responsibilities was a common theme [24, 26, 27, 29, 32]. Some people found it difficult to arrange appointments around busy schedules. This related to worries about taking time off work [29, 30, 32] or having competing health priorities, such as an operation due around the same time as diagnostic testing [30]. Competing health priorities presented as difficulties balancing other health conditions with attending diagnostic testing. For instance, comorbidities and related treatments or physical or mental health problems sometimes prevented appointment attendance [24, 27, 29, 34].

I am being admitted to hospital on December 2^nd^ 2011 for hip replacement otherwise I would have been happy to participate.

(Ali et al. [24])

#### 3.5.4 Lack of Knowledge or Awareness

Lack of awareness or knowledge reduced likelihood of access and was related to the condition itself, benefits of early detection, test practicalities and causal factors [28, 32, 33, 40].

Regarding cervical screening, there was a lack of awareness around testing practicalities, such as testing frequency and age at which the test is offered [33]. In one focus group, poor knowledge about cervical cancer was linked to stigma and fatalism [33]. In another study, one participant stated,

I didn't know anything about cervical cancer and that I had to go for cervical screening … I didn't go because I didn't know that it was important.

(Logan and Macllfatrick [32])

Researchers in Tsipa's [40] study reported, ‘not forming a direct link between screening and early diagnosis of cancer, as well as reduced awareness about the benefits of detecting cancer early, are both factors that can negatively impact on people's decisions to participate in screening’. This supports results discussed in Section 3.3.1, indicating that people who perceive diagnostic testing as beneficial are more likely to access than those who do not.

#### 3.5.5 Healthcare Professionals' Characteristics

Healthcare professionals' personal characteristics could increase or decrease likelihood of access, depending on participant perceptions [32, 33, 35, 36, 40]. According to researchers,

Particularly, participants seemed more likely to seek medical advice from their GPs when they perceived them as friendly, understanding, as taking a positive approach to their concerns and considerate in addressing sensitive healthcare issues.

(Tsipa [40])

Lack of encouragement from healthcare professionals led some to believe that testing was unimportant, discouraging access, while presence of encouragement convinced others to access services when they may not have otherwise.

The last time I went for my medical for work the doctor went, ‘Oh you haven't had a smear for a while. Just go around and get it while you're here.’ I was like, ‘No, I haven't time, no’, and he went, ‘No, you've plenty of time.’ … I did go and afterwards I was sort of like glad I went.

(Logan and Macllfatrick [32])

However, how healthcare professionals encourage access should be considered with sensitivity and care.

I remember, now it's a while ago, but I had to go for my six week check‐up … He says right, while you're here … So it wasn't we'll send for you, but we'll do it. You're here for your six week check, it is being done. So there was no option. I hadn't even my legs shaved or anything or time for a bath, I was mortified.

(Logan and Macllfatrick [32])

#### 3.5.6 Ability to Trust

Ability to trust and develop rapport with healthcare professionals was important to increase likelihood of access [25, 33, 37, 38, 40].

I think that going to the GP would be the easiest for me. They know what they're doing and they do it very efficiently, so I'm quite happy with that idea.

(Brown et al. [25])

In one study, this extended to trust in the NHS.

The best part of this screening, and any screening you do as part of the NHS, if they do find anything that you will get treatment. Because we are very lucky here, we do get treatment, and we have amazing, super‐hero NHS staff and we are well looked‐after.

(Tsipa [40])

Lack of trust was shown to discourage access [37, 40] and ranged from an inability to trust test results to general distrust in the healthcare system [33, 40]. For some, distrust was deeply embedded and appeared to frame overall perceptions of the healthcare system.

… In my mother's day it was hysterectomies. About half the women had hysterectomies, which were unnecessary, right? Nowadays you have these breast mastectomies, and it seems to me that there is a sort of trend that men like carving up women's bodies that isn't necessary. So … I'm sceptical about medical politics, trends, data security, practices, fashions if you like.

(Tsipa [40])

### 3.6 PPI

**Figure 6:**
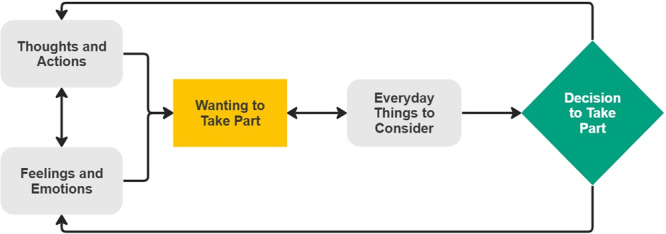
Simplified language conceptual model.

After themes were identified and mapped to the model, a simplified language version of the model (Figure 6) and its component factors were presented to the two groups of LEEs. LEEs within each group corroborated findings from the review, adding and emphasising elements that were important for them.

#### 3.6.1 Digital Exclusion

LEEs over 70 feared being ‘left behind’ by the NHS' digital transformation. It was discussed that NHS providers are introducing new methods for scheduling appointments, including mobile text messages containing hyperlinks to online systems. LEEs discussed how this was not feasible, particularly for people who do not have a smartphone or who are ‘not online’. This factor could fit within the Practical Factors theme but was not discussed in the literature.

#### 3.6.2 Trust in the NHS

During both PPI sessions, LEEs largely felt unable to trust the NHS, aligning with the findings of the review. Importantly, all LEEs in these groups are White British and if other ethnic groups had been present during the discussion, this view may have differed, as indicated by findings related to ethnic minority and migrant participants' perceptions.

#### 3.6.3 Fear of Judgement

In line with the findings of the review, fear of judgement was discussed in both sessions; however, mode of judgement differed between men and women. While men discussed fearing judgement from society and their peers, primarily fearing being labelled as ‘ill’ and the implications of this, women feared judgement from healthcare professionals. Women self‐identifying as ‘overweight’ feared that healthcare professionals would recommend weight loss, rather than attempting to provide a diagnosis. However, even if weight loss was recommended, women reported that little support was offered.

## 4 Discussion

This study aimed to evaluate determinants of accessing diagnostic services for British residents experiencing high deprivation. The conceptual model, as corroborated and supplemented by LEEs' experiences, illustrates the interactive and mediative nature of these determinants.

### 4.1 A Multiplex, Mediative and Iterative Model

Findings from this review indicate that decision‐making around diagnostic service access for people experiencing high deprivation in the United Kingdom is a complex, iterative process that can be mediated by several interactive factors. Where certain facilitators and/or barriers are present, these can be mediated by the presence of others. Fear, for instance, has been shown to have a complex influence on healthcare access [42, 43, 44]. Our model may contribute to simplifying the understanding around the influence of fear on diagnostic service access. As an Emotional and Psychological Factor, fear can initially be mediated by any factor within Beliefs and Behaviours, which will ultimately result in the presence or absence of Motivation to access the services. The absence or presence of Motivation can then be mediated by Practical Factors, which may act as facilitators or barriers to access, resulting in a decision on whether to access. Therefore, fear is only one element of the decision‐making process. While it may be a prominent emotion or psychological state for some, it can be mediated by the presence of other factors. Additionally, fear will not be experienced the same way for everyone. Some people may fear receiving a diagnosis, therefore reducing their Motivation to access diagnostic services, while others may fear suffering, which may increase their Motivation to access the services due to the Belief that early detection may result in less suffering. It is therefore apparent that no single factor acts in isolation. It is the culmination of all factors that determines whether diagnostic services will be accessed.

Additionally, our model illustrates that motivation alone is not sufficient to encourage participation [45]. Even where Beliefs and Behaviours and Emotional and Psychological Factors result in Motivation to access diagnostic services, Practical Factors must also align to enable access.

The model also indicates that the decision‐making process is iterative, which was corroborated by LEEs, who discussed that they would not fear cervical screening unless results were abnormal, at which point they would fear any subsequent testing. This illustrates how the Decision to access diagnostic services influences Emotional and Psychological Factors, beginning the cycle again for any subsequent testing.

Some determinants identified in this review were exclusively experienced by specific groups. Factors such as a sense of civic duty, not wanting to waste NHS resources and valuing the NHS were seen in ethnic minority and migrant groups and were contrasted by opposing views by native and White British participants.

This review included participants from Southeast Asia, North and Central Africa, the Caribbean and Pakistan. It is important that future studies do not overgeneralise the experiences of people with different backgrounds and lived experiences. As such, it is imperative that future studies focus on specific demographics to ensure that variations in lived experience are captured and explored appropriately.

### 4.2 Social Determinants of Health

For LEEs and participants in included studies, determinants of diagnostic service access were not usually linked directly to health, but to wider Social Determinants of Health (SDH) [46]. SDH are non‐medical factors that influence health outcomes [47]. It is well established that a social gradient of health and illness exists, such that higher deprivation is associated with poorer health [12].

Additionally, this review has highlighted the significant influence of behavioural and psychosocial factors on a person's decision to access diagnostic services. What a person believes, how they perceive the world and experiences of those around them and their individual and collective experiences contribute to whether they ultimately access diagnostic services. It is important that these factors, including the social, psychological and behavioural influences on active and passive decision‐making, are understood by policymakers, community‐based decision makers and practitioners when attempting to improve access.

### 4.3 Comparison With Existing Models

There are several existing models for healthcare access, including but not limited to Andersens' Behavioural Model of Health Services Use [48], Penchansky and Thomas' theory of access [49], the COM‐B model [50] and Levesque's Conceptual Framework for Healthcare Access [51]. While these models incorporate elements included in our model (for instance, all mentioned models incorporate some element of behavioural, psychological, social and/or practical factors affecting access), our model illustrates the unique experiences of people experiencing high deprivation in the United Kingdom when deciding whether to access diagnostic services. Whether and how our model compares against existing models, and whether the experiences of people living in high deprivation in the United Kingdom are unique or common amongst other groups should be further explored in future studies.

Until tested for use in other domains, within different populations or regarding different types of healthcare, our model is limited to the uses indicated in this review. Its key unique factor is its illustration that even where barriers exist, these can be overridden by facilitators later down the line, or vice versa. Our model, therefore, illustrates the complexity of decision‐making regarding diagnostic service access, while providing a useful tool for healthcare and community‐based decision makers to identify and address barriers to access and enhance the influence of facilitators.

### 4.4 Strengths and Limitations

Incorporating PPI into this review was essential. The PPI sessions not only corroborated the findings but also emphasised important factors for people experiencing high deprivation in South England specifically. PPI is a useful tool for assessing how synthesisation of existing literature may not capture the finer details of lived experience. Our PPI sample was relatively small and ethnically homogeneous, which may have been a limitation of this review. While the PPI sample in this review was ethnically homogeneous, this was largely expected, because the population on the estate is primarily comprised of White British residents, with a small proportion (6.1% for the whole of the county; specific data for the estate are unavailable) of the population comprising individuals from ethnic minority or migrant backgrounds [52]. No adults living on the estate were excluded from participating in the PPI sessions, unless they did not have the capacity to consent. However, greater efforts could have been made to include people from ethnic minority and migrant backgrounds and any future studies regarding this review should incorporate these efforts.

PPI is not intended to be representative of the wider population, but to assess different perspectives based on the PPI group's lived experience [53]. While the ethnic homogeneity of the PPI group may act as a limitation for this review, the LEEs included in the PPI sessions were not intended to be representative of the area's population. They were included so they could share their personal and collective lived experiences relating to diagnostic service access in their area. Future studies should aim to incorporate perspectives from people with different lived experiences, either in this area or others.

As this review consisted of a systematic, qualitative analysis of the literature, there was an element of subjectivity. Although attempts were made to reduce this through involving a PPI group and communicating and collaborating with the research team, the authors acknowledge that bias and subjectivity cannot be completely removed from qualitative studies. However, the aim of this qualitative research is not to provide objective neutrality [54, 55], but to explore the unique (or common) experiences of those with specific experiences living in a specific area. To reduce any influence of researcher or LEE bias, future studies should assess the applicability of the model in real‐world practice, in other domains and in different parts of the United Kingdom and/or the world, expanding on the findings from this review and contributing to the wider body of knowledge regarding diagnostic service access.

### 4.5 Implications for Policy, Practice and Further Research

It is envisaged that this model can be used in two parts by policymakers, practitioners and community‐based decision makers. First, patients' participation in diagnostic testing can be mapped against the model to understand barriers and facilitators to access for a specific sample. Once understood, tailored interventions can be planned and implemented that aim to address barriers and enhance facilitators. Interventions can target any of the three categories, but, due to the anticipated complexity of real‐world applications of the model, it is suggested that more effective interventions will target more than one.

For instance, an intervention campaign could aim to improve potential patients' perceived benefits of testing, as well as knowledge and awareness of causal factors and the conditions themselves, particularly for those less often highlighted by the media. NHS trusts could simultaneously emphasise healthcare professionals' demeanour by offering specific training to encourage the fostering of trust. Services could also be offered in a more convenient and familiar location, as is currently being demonstrated through the Community Diagnostic Centre programme, to reduce the likelihood that competing priorities will act as barriers to participation. This could also help to address some practical barriers, such as travel difficulties. Any proposed interventions should make use of community members' voice and emphasise the value of lived experience expertise. Efforts should also be made to emphasise the influence of facilitators, rather than simply reducing the influence of barriers. This could include incorporating community‐based sessions, led by trusted members within the community, to encourage potential patients to discuss any concerns or fears, as well as hopes, in accessing diagnostic services.

In included studies, determinants of diagnostic access tended to be measured through recruitment of patients already registered with a General Practitioner and therefore eligible to receive a diagnostic invitation. This excludes people who may not access General Practitioner services for any reason. To address this gap, future studies should explore whether and how people who are not current healthcare patients access diagnostic services. Additionally, the authors found that determinants of access varied based on ethnic background and gender. Future studies should explore what differences may be present amongst different groups and why people perceive these differences to exist.

Future studies should also explore whether the model and its component themes and sub‐themes are comparable to factors affecting access in other high‐income countries.

Finally, future studies should both test our model's usefulness with other samples and evaluate whether it can be extrapolated and used in other contexts.

## 5 Conclusion

By emphasising participants' lived experience, this review has illustrated the complexity associated with diagnostic service access, particularly when described directly by qualitative study participants and LEEs. The conceptual model proposed in this review illustrates this complexity, as well as the mediative, interactive and iterative nature of deciding to access diagnostic services for people experiencing high deprivation in the United Kingdom. No single factor acts in isolation. It is the culmination of all elements experienced by the person deciding whether to access diagnostic services that determine whether they ultimately access, and continue to access, these services.

The proposed model should be applied in policy and practice to enable understanding of the factors influencing access to diagnostic services and to design interventions that address identified determinants.

## Author Contributions

**Christine Vincent:** conceptualisation, investigation, writing–original draft, methodology, visualisation, formal analysis. **Lee‐Ann Fenge:** supervision, writing–review and editing, funding acquisition, formal analysis. **Sam Porter:** writing–review and editing, supervision, formal analysis. **Sharon Holland:** writing–review and editing, supervision, formal analysis.

## Conflicts of Interest

The authors declare no conflicts of interest.

## Data Availability

Data sharing is not applicable to this article as no new data were created or analysed in this study.

## Appendix A

See Table A1.

**Table A1:** ENTREQ Guidelines Checklist (adapted from Tong et al. [18]).

Item	Guide and description	Reported on
Aim	State the research question that the synthesis address.	1
Synthesis methodology	Identify the synthesis methodology or theoretical framework that underpins the synthesis and describe the rational for choice of methodology (e.g., meta‐ethnography, thematic synthesis, critical interpretive synthesis, grounded theory synthesis, realist synthesis, meta‐aggregation, meta‐study and framework synthesis).	2
Approach to searching	Indicate whether the search was preplanned (comprehensive search strategies to seek all available studies) or iterative (to seek all available concepts until theoretical saturation is achieved).	2.2
Inclusion criteria	Specify the inclusion/exclusion criteria (e.g., in terms of population, language, year limits, type of publication and study type).	2.3
Data sources	Describe the information sources used (e.g., electronic databases [MEDLINE, EMBASE, CINAHL, PsycINFO and Econlit], grey literature databases [digital thesis, policy reports], relevant organisational websites, experts, information specialists, generic web searches [Google Scholar], hand searching and reference lists) and when the searches were conducted; provide the rationale for using the data sources.	2.2
Electronic search strategy	Describe the literature search (e.g., provide electronic search strategies with population terms, clinical or health topic terms, experiential or social phenomena–related terms, filters for qualitative research and search limits).	2.2.1
Study screening methods	Describe the process of study screening and sifting (e.g., title, abstract and full‐text review and number of independent reviewers who screened studies).	2.4
Study characteristics	Present the characteristics of the included studies (e.g., year of publication, country, population, number of participants, data collection, methodology, analysis and research questions).	3.3
Study selection results	Identify the number of studies screened and provide reasons for study exclusion (e.g., for comprehensive searching, provide numbers of studies screened and reasons for exclusion indicated in a figure/flowchart; for iterative searching, describe reasons for study exclusion and inclusion based on modifications to the research question and/or contribution to theory development).	3.1
Rationale for appraisal	Describe the rationale and approach used to appraise the included studies or selected findings (e.g., assessment of conduct [validity and robustness], assessment of reporting [transparency], assessment of content and utility of the findings).	2.5
Appraisal items	State the tools, frameworks and criteria used to appraise the studies or selected findings (e.g., Existing tools: CASP, QARI, COREQ and Mays and Pope (2000); reviewer‐developed tools; describe the domains assessed: research team, study design, data analysis and interpretations and reporting).	2.5
Appraisal process	Indicate whether the appraisal was conducted independently by more than one reviewer and if consensus was required.	2.5
Appraisal results	Present results of the quality assessment and indicate which articles, if any, were weighted/excluded based on the assessment and give rationale.	2.5
Data extraction	Indicate which sections of the primary studies were analysed and how were the data extracted from the primary studies? (e.g., all text under the headings ‘results/conclusions’ was extracted electronically and entered into a computer software).	2.4
Software	State the computer software used, if any.	2.4
Number of reviewers	Identify who was involved in the coding and analysis.	2.4
Coding	Describe the process for coding the data (e.g., line‐by‐line coding to search for concepts).	2.6
Study comparison	Describe how were comparisons made within and across studies (e.g., subsequent studies were coded into pre‐existing concepts, and new concepts were created when deemed necessary).	2.6
Derivation of themes	Explain whether the process of deriving the themes or constructs was inductive or deductive.	2.6
Quotations	Provide quotations from the primary studies to illustrate themes/constructs and identify whether the quotations were participant quotations or the author's interpretation.	3.3
Synthesis output	Present rich, compelling and useful results that go beyond a summary of the primary studies (e.g., new interpretation, models of evidence, conceptual models, analytical framework and development of a new theory or construct).	3.3
